# The challenge of diagnosing *Plasmodium ovale *malaria in travellers: report of six clustered cases in french soldiers returning from West Africa

**DOI:** 10.1186/1475-2875-9-358

**Published:** 2010-12-10

**Authors:** Franck de Laval, Manuela Oliver, Christophe Rapp, Vincent Pommier de Santi, Alexandre Mendibil, Xavier Deparis, Fabrice Simon

**Affiliations:** 1 Department of Epidemiology and Public Health, Tropical Medicine Institute of the Military Health Service, Marseille, France; 2 Department of Infectious Diseases and Tropical Medicine, Laveran Military Teaching Hospital, Marseille, France; 3 Department of Infectious Diseases and Tropical Medicine, Begin Military Teaching Hospital, Saint-Mandé, France; 4 Cabinet Médical, 8RPIMa, Castres, France

## Abstract

**Background:**

*Plasmodium ovale *is responsible for 5% of imported malaria in French travellers. The clinical and biological features of six clustered cases of *P. ovale *malaria in an army unit of 62 French soldiers returning from the Ivory Coast are reported.

**Case report:**

All patients were symptomatic and developed symptoms on average 50 days after their return and 20 days after the end of chemoprophylaxis (doxycycline). Clinical features included fever (6/6), mostly tertian (4/6), aches (6/6), nausea (3/6), abdominal pain (2/6), diarrhoea (2/6), or cough (2/6). Thrombocytopaenia was lower than 100,000/mm^3 ^in half the cases only, and the haemoglobin count was normal for all patients. The diagnosis was made after at least three thick and thin blood smear searches. Parasitaemia was always lower than 0.5%. All rapid diagnostic tests were negative for HRP2 and pLDH antigens.

**Discussion:**

*Plasmodium ovale *malaria is currently a problem to diagnose in travellers, notably in French soldiers returning from the Ivory Coast. Early attempts at diagnosis are difficult due to the lack of specific clinical features, the rarity of biological changes and the poor sensitivity of diagnostic tools to detect low parasitaemia. Thus, the diagnosis is commonly delayed or missed. Physicians should be aware of this diagnostic challenge to avoid relapses and provide prompt and adequate treatment with chloroquine and radical cure with primaquine.

## Background

Malaria is a threat to travellers staying in tropical endemic areas, including soldiers under field conditions [1,2]. Each year, 40,000 French soldiers stay in or go through malaria-infected areas, using mandatory mosquito-fighting measures and chemoprophylaxis (currently doxycycline). In 2006, the French Army experienced 557 cases of malaria, 60% of which were due to *Plasmodium falciparum *[3]. About half of these malarial attacks were diagnosed in soldiers after their return from endemic areas [3]. Continuous monitoring identified a recent increase in imported non-falciparum malaria, mainly *Plasmodium vivax *from French Guiana and *Plasmodium ovale *from West Africa [3,4]. This is a report of six clustered cases of imported *P. ovale *malaria in French soldiers who were infected in the Ivory Coast, pointing out the difficulties in diagnosing this species.

## Cases report

Six cases of *P. ovale *malaria were successively diagnosed among French soldiers returning from the Ivory Coast. They all belonged to the same army unit (62 people), which had stayed in Abijdan, Bouake and Yamoussoukro between 01/10/2007 and 15/04/2008. During the mission, this unit suffered 16 cases of fevers of unknown origin (two of them included in the cases reported here), but no malarial attack confirmed by rapid antigenic tests. An epidemiological study with behavioural questionnaire was conducted in the unit among the cases and uninfected soldiers. The incidence rate for malarial infection was 6/62 (10%) over 6.5 months. All the patients admitted having imperfectly used anti-mosquito measures, but all declared having correctly taken doxycycline chemoprophylaxis during the mission and after their return. No behavioural factors were statistically associated with the occurrence of malaria in this small group.

All infected cases were men (mean age: 32 years, range: 30-36). The first symptoms developed on average 50 days after their return (range: 41-72) and an average of 20 days after the end of chemoprophylaxis (range: 11-42). The patients consulted on average two days after the onset of symptoms. The most frequent symptoms were fever (6/6), mostly tertian (4/6) with temperatures higher than 38.5°C in half the cases, aches (6/6) and shivers (5/6). Other symptoms included nausea (3/6), abdominal pain (2/6), diarrhoea (2/6), or cough (2/6). No patient had a splenomegaly. Biological changes (Table 1) included thrombocytopaenia lower than 100,000/mm^3 ^(3/6), hepatic cytolysis (3/6), hyperbilirubinaemia (1/6), and elevated CRP (6/6). The haemoglobin count was normal for all patients.

**Table 1:** Delays and biological changes in 6 French soldiers with imported Plasmodium ovale malaria.

Patient	1	2	3	4	5	6
Number of days between end of chemoprophylaxis and disease onset	19	18	11	12	42	17
Number of days between onset and diagnosis	8	5	12	11	3	7

Haemoglobin (g/dL) *[Normal = 13-18 g/dL]*	13.2	13.0	13.9	15.1	14.1	15.3
White blood count (x 10^3^/mm^3^) *[Normal = 4-10 × 10^3^/mm^3^]*	6.6	5.7	5.4	2.0	8.5	5.9
Blood platelets (x 10^3^/mm^3^) *[Normal = 150-400 × 10^3^/mm^3^]*	147.0	97.0	297.0	60.0	170.0	60.0
CRP (mg/L) *[Normal < 10 mg/L]*	24.0	27.0	50.0	122.0	25.0	118.0
Creatinaemia (μmol/L) *[Normal = 70-100 μmol/L]*	79.0	90.0	103.0	120.0	73.0	88.0
Glucose (mmol/L) *[Normal = 4-7.25 mmol/L]*	5.3	6.1	6.3	6.5	4.5	6.8
ASAT (UI/L) *[Normal = 5-40 UI/L]*	27.0	23.0	89.0	34.0	24.0	91.0
ALAT (UI/L) *[Normal = 5-35 UI/L]*	34.0	16.0	129.0	96.0	40.0	179.0
Bilirubin (mg/L) *[Normal < 12 mg/L]*	7.8	43.0	16.0	9.0	10.0	11.0
Haptoglobin (g/L) *[Normal = 1-3 g/L]*	1.1	0.21	1.6	1.0	1.2	0.3

The diagnosis was established using thick and thin blood smears, after at least three searches for each case (up to seven times for one case), and on average eight days after the onset of symptoms (range: 3-12). Parasitaemia was always lower than 0.5%. Rapid diagnostic tests were all negative both for the HRP2 antigen (specificity for *P. falciparum*) and for the pLDH antigen (specificity for *Plasmodium spp*, thus for *P. ovale*).

Three patients received chloroquine (25 milligrams/kilo within three days). Three patients received quinine (24 mg/kg/d for seven days) because of suspected co-infection with *P. falciparum *(two cases) or digestive intolerance (one case treated by intravenous quinine). All attacks were cured. One patient experienced a *P. ovale *relapse 30 days after his first attack. He received a further course of chloroquine. All patients also received a course of primaquine (15 mg/d for 14 days) to avoid malarial relapse, none having a glucose-6-phosphate-dehydrogenase (G6PD) deficiency. No new relapse was reported after a 24-month follow-up.

## Discussion

For travellers, *P. falciparum *is the main target of malarial prevention because this species is widely endemic, very common and clinically severe [1]. Although very rarely life-threatening, relapsing malaria species, *P. vivax *and *P. ovale*, are also of concern for travellers in endemic areas. In contrast to *P. vivax*, which is widespread in Asia and Central and Southern America, *P. ovale *is only endemic in some African countries [5]. Two subspecies of *P. ovale *have been identified in West Africa [6]. *Plasmodium ovale *is responsible for rare, travel-acquired infection [7]. However, the French Army experienced a significant increase in the incidence rate of *P. ovale *malaria attacks among French soldiers returning from the Ivory Coast between 2002 and 2007, but not among soldiers in the field, while the incidence of *Pl. falciparum *regularly decreased over the same period [3,4]. These changes could reflect epidemiological changes in the local transmission of malaria and/or a lower effectiveness of doxycycline chemoprophylaxis on *P. ovale *than on *P. falciparum*. Indeed, *P. ovale *was very uncommon while using the association chloroquine-proguanil in the French Army before 2002. Thus, preventing *P. ovale *infection with doxycycline seems challenging because of the lack of action on hypnozoites. A similar failure has also been recently reported with atovaquone plus proguanil chemoprophylaxis [8].

Diagnosing *P. ovale *infection in infected patients is a challenge in the field or on return to non-endemic countries [9]. Symptoms can be due to a primary infection or, later to relapses that occur in one patient in ten because of delayed parasitaemia from intrahepatic dormant forms [1]. Shortening the time-lapse before effective treatment of this relapsing malaria is required to reduce morbidity, limit sick-leave and, diminish the risk of spleen rupture [10]. This experience illustrates a few traps that physicians should be aware of when attempting a diagnosis. First, all clinical features of *P. ovale *attacks are not specific. Nevertheless, a tertian fever-evocative although non-specific - was present in more than half the patients, possibly due to the time lapse to the delayed diagnosis, which had allowed synchronization of the replication cycle. Moreover, digestive or respiratory symptoms, which were present in half cases, can wrongly suggest a viral infection. However, any fever, associated or not, after return from an endemic area, should be considered to be due to malaria and be followed up by immediate thick and thin blood smears. As a result of health education before and during the mission, all the patients promptly consulted a general practitioner and underwent a search for malaria in a nearby laboratory. In contrast to *Plasmodium falciparum *and *Plasmodium vivax *malaria, the haemogram appeared not to be very helpful for *Plasmodium ovale *because anaemia is frequently missing and thrombocytopaenia mild or absent [7], especially within the first days of the attack. Biological markers for haemolysis were also missing in five cases out of six. Most of all, the sensitivity of routine microscopic searches for *P. ovale *in imported cases is very low [11], as shown in this series. Although more pyrogenic than *P. falciparum *[12], the level of parasitaemia for *P. ovale *is far lower at the same time after disease onset [7]. Unfortunately, all available rapid antigenic tests currently lack sensitivity to *P. ovale*, while they are highly sensitive to *P. falciparum *[13-15]. They generally fail to detect *P. ovale *infection when thick films and blood smears are negative. This could be due to the very low level of circulating antigen or to inadequate antigens used for these tests. This could explain some acute unexplained fevers in the French Army in the field in Africa [3]. When the diagnosis of imported *P. ovale *malaria is suspected, routine microscopic searches with thick and thin blood smears should be repeated, up to three times and in an expert laboratory, if possible [9]. Considering the high sensitivity and specificity of molecular detection of *P. ovale *using PCR, this tool marks real progress in confirming the diagnosis, although it is still not routinely available [5,7,16]. It can be used as a second-line diagnosis tool to identify infra-microscopic parasitaemia, especially for unexplained relapsing fever in travellers.

The treatment for proven attacks is based on chloroquine (25 mg/kg for three days). The treatment against dormant stages in the liver consists of a radical cure with primaquine (0.5 mg/kg/d for 14 days) in patients without G6PD deficiency [17,18]. Failures of primaquine are unusual, mostly due to poor observance or inadequate dosage [19].

To date, diagnosing *P. ovale *infection in travellers returning from endemic areas is still a challenge for physicians and requires repeat microscopic searches to detect low parasitaemia. As PCR is not a routine tool, there is a real need to improve the sensitivity of rapid diagnostic tests for this plasmodial species.

## Abbreviations

FL: participated in the care of the patients, in the epidemiological study and drafted the manuscript; CR: participated in the care of the patients and drafted the manuscript; MO: participated in the care of the patients; VPS: participated in the epidemiological study; AM: participated in the care of the patients; XD: participated in the epidemiological study; FS: participated in the care of the patients and drafted the manuscript.

## Competing interests

The authors declare that they have no competing interests.

## Authors' contributions

All authors read and approved the final manuscript.
